# Ethnic Differences in Bone Health

**DOI:** 10.3389/fendo.2015.00024

**Published:** 2015-03-17

**Authors:** Ayse Zengin, Ann Prentice, Kate Anna Ward

**Affiliations:** ^1^Medical Research Council Human Nutrition Research, Cambridge, UK; ^2^Medical Research Council, Keneba, Gambia

**Keywords:** ethnic groups, bone, muscle, pQCT, DXA, fracture, skeletal, bone mineral density

## Abstract

There are differences in bone health between ethnic groups in both men and in women. Variations in body size and composition are likely to contribute to reported differences. Most studies report ethnic differences in areal bone mineral density (aBMD), which do not consistently parallel ethnic patterns in fracture rates. This suggests that other parameters beside aBMD should be considered when determining fracture risk between and within populations, including other aspects of bone strength: bone structure and microarchitecture, as well as muscle strength (mass, force generation, anatomy) and fat mass. We review what is known about differences in bone-densitometry-derived outcomes between ethnic groups and the extent to which they account for the differences in fracture risk. Studies are included that were published primarily between 1994 and 2014. A “one size fits all approach” should definitely not be used to understand better ethnic differences in fracture risk.

## Introduction

There are differences in fracture risk between ethnic groups in both men and in women across the globe. It is important to understand the underlying phenotype that contributes to these differences in order to be able to determine strategies for the prevention of osteoporosis and bone fragility in an ever-changing environment. Most studies report ethnic differences in areal bone mineral density (aBMD), which do not consistently parallel ethnic patterns in fracture rates. Variations in body size and composition are likely to contribute to reported differences.

Traditionally, aBMD (g/cm^2^) measured by single and dual energy x-ray absorptiometry (DXA) or photon absorptiometry (SPA/DPA) is used as a surrogate for bone strength and fracture risk. DXA superseded SPA/DPA over 30 years ago. DXA has many advantages, including good precision, low radiation dose, and ability to scan skeletal sites most prone to osteoporotic fracture. Areal BMD predicts fracture and in Western White older adults, a 1-SD decrease in aBMD leads to 2.6-fold greater fracture risk (1). However, DXA only measures aBMD (g/cm^2^), projectional bone area (cm^2^), and bone mineral content (BMC, g) and as such does not fully account for bone size because it cannot account for differences in bone depth. Additionally, DXA is an average value across all bony elements within the periosteal envelope and so does not assess the separate compartments within bone (cortical or trabecular), bone structure (size and shape), organization within the periosteal envelope (e.g., cortical thickness), microarchitecture (e.g., trabecular thickness, number, cortical porosity), or bone metabolism, all of which are important components of bone strength. The aforementioned limitations are thought to be the basis for the inadequacies of aBMD in predicting fracture in different ethnic groups (2). The advancement of imaging technology to include central, peripheral, and high-resolution peripheral quantitative computed tomography (QCT, pQCT, and HRpQCT) has enabled the measurement of volumetric bone mineral density (vBMD), the structural dimensions, and internal organization of cortical and trabecular bone and to give *in vivo* estimates of bone strength. With its greater spatial resolution (82 μm), HRpQCT also measures trabecular and cortical microarchitecture; it measures trabecular number directly and provides indirect estimates of trabecular bone volume, trabecular thickness, trabecular separation, and bone strength (3).

Throughout this review, ethnicity has been used to group individuals according to a mix of cultural and other factors including geography, language, diet, religion, ancestry, and physical features traditionally associated with race. The glossary in Table 1 has summarized and defined each ethnic group that is discussed in this review.

**Table 1:** **Classification of ethnic groups**.

Ethnic group	Definition
White-American	European ancestral origins living in US
Black-American	African ancestral origins living in US
Asian-American	Collective group of eastern Asian (Japanese, Chinese) ancestral origins living in US
Chinese-American	Chinese ancestral origins living in US
Japanese-American	Japanese ancestral origins living in US
White-British	English, Welsh, Scottish, and Northern Irish living in UK
South-Asian British	Indian, Pakistani, and Bangladeshi ancestral roots living in UK
Afro-Caribbean British	African ancestral origins whose forebears were in the Caribbean before immigrating to UK
Gambian	Sub-Saharan African ancestral origins, primarily Mandinka, living in The Gambia
Gambian-British	Sub-Saharan African ancestral origins, primarily Mandinka, living in UK
Chinese	Chinese ancestral origins living in China

This review focuses on evidence from studies using bone-densitometry techniques and those reporting fracture incidences. With the aforementioned technical developments and the recognition of the limitations of DXA, recent studies have focused on describing ethnic differences in bone structure and bone microarchitecture and the extent to which these may contribute to differences in fracture risk. However, as this review highlights, there are still relatively few data available outside of US, particularly in populations where fracture incidence is predicted to rise over the coming decades.

## Fracture Incidence and BMD Across the Globe

**Figure 1:**
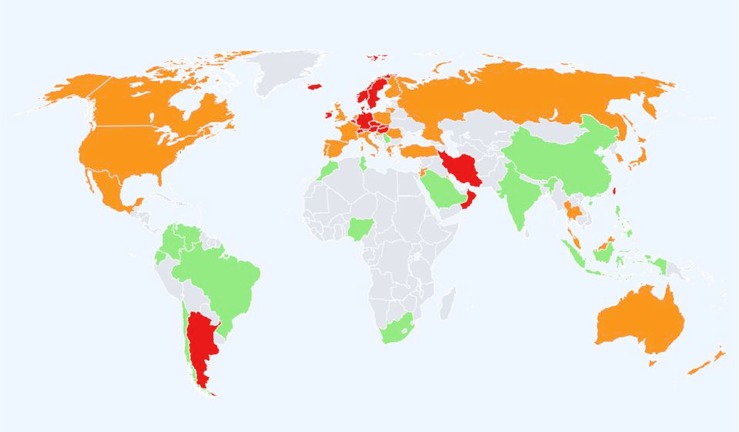
**Hip fracture rates for men and women combined in different countries of the world categorized by risk**. Where estimates are available, countries are color coded red (annual incidence >250/100,000), orange (150–250/100,000), or green (<150/100,000) (6).

Global data on adults suggest that, compared to age-matched White-American or British/European populations, other ethnic groups have a lower incidence of fracture (Figure 1). There is a >10-fold variation in age-standardized hip fracture risk across 63 countries and a notable divide between Western and Eastern populations (4–6). Most recently, Cauley et al. (5) showed greater variability in incidence and geographic pattern for clinical vertebral fractures than for hip fracture; it should be noted that for radiographically confirmed vertebral fractures the global pattern incidence was similar to that for hip fracture (5). Globally, the lowest fracture rates are in populations with African ancestry (6), but there is a sparcity of data from the African continent, particularly Sub-Saharan Africa (5, 7, 8). In the late 1960s, Solomon et al. described differences between White and Bantu populations in adult fracture incidence to be similar to those reported in North-America between Black- and White-Americans (9). Data from Cameroon suggest similarities to populations from the developed world because women have higher incidence than men of low trauma fracture to the hip and wrist (10). Further data are required to confirm the generalizability of this observation. With a rising life expectancy, increasing “Westernization” of African populations, better survival for individuals with HIV, and increasing non-communicable disease risk fracture incidence is also expected to rise and should be better characterized (5, 7, 10, 11). In Asian populations, a 15-fold increase in hip fracture incidences was reported in studies from Japan and Hong Kong (12, 13). Compared to Western populations, there also appear to be sex differences in the patterns of incidence where in China there were no sex differences and in Thailand, men have greater hip fracture incidence (14, 15).

To date, the majority of large population studies have used aBMD measured by DXA as a proxy for fracture risk to study ethnic differences in bone health. In men, data from the third National Health and Nutrition Examination Survey (NHANES III) showed that Black-Americans had a higher mean femoral neck and total hip aBMD compared to White-American men (16); these differences were similar to reported fracture rates between these ethnic groups (17). The osteoporotic fractures in men study (MrOS) used QCT and showed that Black-American and East Asian-American men had higher bone strength compared to White-American men due to greater vBMD at the femoral neck (18). Data from UK-cohort of the European Male Aging Study (EMAS) compared White-British men to a group of Afro-Caribbean British and South-Asian British men. The Afro-Caribbean British group had higher aBMD at all sites compared to South-Asian British and White-British, both before and after adjustment for body size (19). However, vBMD of the distal radius assessed using pQCT was not different between the groups (20).

One of the largest multi-ethnic studies, the National Osteoporosis Risk Assessment (NORA) study (NORA) showed that Black-American women had higher, and Asian-Americans^1^ lower, aBMD in the study population (2). Even after adjusting for body weight and other risk factor covariates, the greater aBMD in Black-American women persisted; however, Asian-American women had similar values to White-American women (2). Despite lower aBMD in Asian-American women, studies have shown lower rates of fracture compared to White-American women (2). In UK, South-Asian women had lower aBMD than White-British women before appropriate adjustment for body or bone size, thereafter there were no differences between groups (21, 22). Similarly, Chinese women had lower aBMD than White-British women; after adjustment for body size, these differences were attenuated (23). Furthermore, despite a lower size-adjusted BMC at the lumbar spine, hip, and radius in Gambian women compared to White-British women (independent of height and weight), there was a lower incidence of fracture among Gambian women (24), suggesting that there are other factors contributing to the lower fracture risk seen in Gambian women. Taken together, all of the studies discussed in this section suggest that there may be other components of bone strength that should be studied to help to better predict fracture risk in different populations.

## Ethnic Differences in Bone Structure

Earlier studies of bone structure show differences in body-segment proportions (sitting and standing height) and also in cortical parameters measured using histomorphometry, which may contribute to ethnic differences in fracture rates (25, 26). The most recent studies also demonstrate that bone structure and microarchitecture are different in ethnic groups and these variations are likely to contribute to the ethnic differences in fracture rates.

### Dual energy x-ray absorptiometry

The Study of Womens Health Across the Nation (SWAN) study reported no differences in aBMD between Chinese-American, Japanese-American, and White-American women, but bone structure varied greatly (27). Femoral neck cross-sectional area (total bone surface in a cross-section, exclusive of soft tissue spaces and pores) and section modulus of the hip, measured by DXA, were higher in Japanese-American compared to White-American women (27). A greater section modulus in Japanese-American women would give better resistance to axial compressive and bending stresses. In the previously described studies of Gambian and Chinese women, compared to White-British women, ethnic differences in hip axis length (HAL) have been reported, in addition to the aBMD differences (23, 28, 29). Of the three groups, Gambian women had the shortest HAL after correction for height. Similarly, in a study in British men, HAL was shorter in Afro-Caribbean British men compared to White-British and South-Asian British men (19); similar results were reported in Gambian-British compared to White-British men after correcting for weight and height (28). A longer HAL has been associated with a higher risk of hip fracture, and is considered to be a risk predictor for hip fracture (30). And so, it is likely that these structural differences, in part, explain differences between ethnic groups of hip fracture incidence (28, 31).

### Quantitative computed tomography

In UK, there are only sparse data on bone structure measured by axial or peripheral QCT. Pre-menopausal South-Asian British women had lower cortical vBMD, BMC, and thinner cortices at the radial diaphysis compared to White-British women; this was independent of age, height, and weight (32). Despite lower BMC in the South-Asian British women, bone strength, as estimated using the strength strain index (SSI) was similar. This suggests that bones of pre-menopausal South-Asian British women may be efficiently adapted to a lower BMC as a result of a different distribution of bone mineral within the periosteal envelope, thereby preserving bone strength (32, 33). In contrast, another study has shown that post-menopausal South-Asian British women have lower SSI and fracture load at the radius and tibia despite having thicker cortices and higher vBMD; bone cross-sectional area was smaller (34).

In MrOS, Black-American and Asian-American men had thicker cortices, measured using axial QCT, than White-Americans, which led to greater bone strength at the hip (18). Similarly, greater estimated bone strength was found in Afro-Caribbean British and South-Asian British men compared to White-British men. Lower strength in the White-British men was due to smaller bone size at the mid-shaft radius (20).

### High-resolution pQCT

Due to the relatively recent introduction of HRpQCT, there are only limited data using this technology. In a comparison of pre-menopausal women from US, Chinese-American women were shown to have smaller bones, with higher cortical vBMD and different trabecular microarchitecture than White-American women (35, 36). They appeared to have a structural advantage in their trabecular microarchitecture compared to White-American women, with larger, more plate-like trabecule, and a greater plate-rod junction density, which indicates the number of trabecular network connections (35). As plate-like structure contributes to a greater proportion of bone strength than the rods, these structural differences may explain the lower fracture risk in this group of women. Currently, there are no other data using HRpQCT to compare differences in bone structure. Lower fracture risk in African-American women is likely due to thicker cortices and better trabecular microarchitecture, both of which would be reflected in higher aBMD (a composite measurement of trabecular and cortical bone) previously reported in this ethnic group (37).

## Ethnic Differences in Body Composition

There are major differences in body habitus (fat mass, muscle mass, height, and weight) between ethnic groups (Table 2), which should be considered when studying differences in bone between and within different ethnic groups (38). For example, Chinese-American and Gambian women have a low BMI, which is a risk factor for fracture (Table 2) (2, 24). In post-menopausal women, a higher body weight is considered to be protective against fracture due to increased load (from greater muscle and fat mass) on the skeleton and also with fat accumulation providing padding during a fall (39). This is indicated in Black-American women who have greater fat and lean mass and aBMD, and lower fracture risk, compared to White-American women (40) (Table 2). A recent meta-analysis reported that muscle mass exerts a greater positive influence on aBMD than fat mass in men and women combined (41). However, increased weight appears to provide only partial protection, with an audit of fracture cases in UK and results from the Global Longitudinal Study of Osteoporosis in Women showing that obesity was a risk factor for fracture (42, 43). Those who fractured did not have a low aBMD (43), indicating the insensitivity of aBMD to predict fracture.

**Table 2:** **Ethnic differences in body composition in women and men**.

		Chinese-American	South-Asian British	Black-American	Tobago	South-African	Mexican-American
Women	Body weight	↓	↑	↑	↑	=	=
	Height	↓	↓	=	↑	↓	↓
	BMI	↓	↑	↑	↑	↑	↑
	Fat mass	↓	↑	↑			↑
	Muscle mass	↓	↓	↑			↓
Men	Body weight	↓^a^	↓	=	=	↓	↓
	Height	↓^a^	↓	=	=	↓	↓
	BMI	=^a^	=	=	↓	↓	=
	Fat mass	↑^a^	↑^b^	↓	↓		↓
	Muscle mass		↓^b^	↑			
Reference		(23, 50)	(32, 34, 51, 52)	(40, 53)	(45, 54, 55)	(56, 57)	(40, 58)

*All parameters are compared to White individuals*.

*^a^Chinese ancestral origins living in China*.

*^b^South-Asian ancestral origins living in New Zealand*.

The NORA and SWAN studies (2, 44) emphasized the importance of taking into account body weight when quantifying differences in aBMD between Asian-American (Chinese and Japanese) and White-American women. Correcting for body weight decreased or reversed the differences in the total hip or spine aBMD between Asian-American (Chinese and Japanese) and White-American women (2, 44, 45). Similarly, correcting for body size attenuated differences in aBMD at the hip, lumbar spine, and total body in South-Asian British (Pakistani and Gujarati) women compared to White-British women (22, 46).

There is a gap in the literature regarding the differences in body composition between men of different ethnicities (Table 2). The studies mentioned in Table 2 demonstrate that there are differences in muscle mass between ethnic groups. These studies also show that it is important to interpret aBMD from DXA with caution if bone area, height, and weight (or its component parts) are not adequately accounted for, as it may lead to misinterpretation of results (46).

## Ethnic Differences in the Muscle–Bone Relationship

Muscle strength is a composite term that describes muscle mass and anatomy (including intramuscular fat accumulation), force generating capacity and power (33). Muscle contractions are considered as the primary source of load upon the skeleton, more so than body weight, even in weight bearing bones (47). Given the differences in lean mass discussed in the previous section, it is likely that muscle force and power will also be different between ethnic groups, which are likely to contribute to differences in vBMD, size, shape, and distribution of bone. Measurements of muscle mass or cross-sectional area incompletely represent changes in muscle strength (48). Techniques to quantify the functional aspects of muscle strength include grip force, jumping mechanography, dynamometry, and electromyography. These dynamic tests give more accurate estimations of muscle strength than using muscle mass alone (46). Preliminary data from the EMAS demonstrated ethnic differences in the relationship between muscle force and power and age (20, 49).

There is a gap in the literature, because no studies published to date have investigated the relationships of muscle mass and function with vBMD, bone size and shape, and/or microarchitecture between ethnic populations. Differences in muscle force and power may partly underlie the ethnic differences in fracture incidence.

## Conclusion

Fracture incidence varies between ethnic groups. Despite its many advantages, it is now recognized that the variance in fracture rates between ethnic groups cannot solely be explained by DXA. The studies described in this review highlights that “one size does not fit all” and there is a need to understand differences in skeletal phenotype at different stages of life (e.g., pre- versus post-menopausal) and in different populations both within and across continents (33). The separate measurement of cortical and trabecular bone compartments, and bone shape, size, microarchitecture, and metabolism is important to fully understand the differences of bone strength between ethnic groups. This requires technologies that move beyond measurements aBMD/BMC by DXA and use of three-dimensional imaging devices like pQCT and HRpQCT. The investigation of muscle and bone relationships in ethnic groups is also critical to the development of more effective strategies to improve musculoskeletal health and function. Future studies are required to identify how differences in nutrition, cultural preferences, socioeconomic factors, sunshine exposure, and physical activity levels affect bone health between ethnic groups.

## Conflict of Interest Statement

The authors declare that the research was conducted in the absence of any commercial or financial relationships that could be construed as a potential conflict of interest.

## Footnotes

^1^Residing in US, i.e., predominantly East Asian population

## References

[1] Marshall D Johnell O Wedel H. Meta-analysis of how well measures of bone mineral density predict occurrence of osteoporotic fractures. BMJ (1996) 312:1254–9. 10.1136/bmj.312.7041.1254 8634613 PMC2351094

[2] Barrett-Connor E Siris ES Wehren LE Miller PD Abbott TA Berger ML Osteoporosis and fracture risk in women of different ethnic groups. J Bone Miner Res (2005) 20:185–94. 10.1359/JBMR.041007 15647811

[3] Bouxsein ML Bone structure and fracture risk: do they go arm in arm? J Bone Miner Res (2011) 26:1389–91 10.1002/jbmr.442 21695731

[4] Ballane G Cauley JA Luckey MM Fuleihan Gel H. Secular trends in hip fractures worldwide: opposing trends East versus West. J Bone Miner Res (2014) 29:1745–55. 10.1002/jbmr.2218 24644018

[5] Cauley JA Chalhoub D Kassem AM Fuleihan Gel H. Geographic and ethnic disparities in osteoporotic fractures. Nat Rev Endocrinol (2014) 10:338–51. 10.1038/nrendo.2014.51nrendo.2014.51 24751883

[6] Kanis JA Oden A McCloskey EV Johansson H Wahl DA Cooper C. A systematic review of hip fracture incidence and probability of fracture worldwide. Osteoporos Int (2012) 23:2239–56. 10.1007/s00198-012-1964-3 22419370 PMC3421108

[7] Prentice A Diet, nutrition and the prevention of osteoporosis. Public Health Nutr (2004) 7:227–43 10.1079/PHN2003590 14972062

[8] Prentice A Ward KA Schoenmakers I Goldberg GR, editors. Bone Growth in African Children and Adolescents. Boca Raton, FL: Taylor & Francis Group (2012).

[9] Solomon L Osteoporosis and fracture of the femoral neck in the South African Bantu. J Bone Joint Surg Br (1968) 50:2–13. 5641595

[10] Zebaze RM Seeman E. Epidemiology of hip and wrist fractures in Cameroon, Africa. Osteoporos Int (2003) 14:301–5. 10.1007/s00198-002-1356-1 12730790

[11] Cooper C Campion G Melton LJ III. Hip fractures in the elderly: a world-wide projection. Osteoporos Int (1992) 2:285–9. 10.1007/BF01623184 1421796

[12] Tsang SW Kung AW Kanis JA Johansson H Oden A. Ten-year fracture probability in Hong Kong Southern Chinese according to age and BMD femoral neck T-scores. Osteoporos Int (2009) 20:1939–45. 10.1007/s00198-009-0906-1 19326036

[13] Orimo H Yaegashi Y Onoda T Fukushima Y Hosoi T Sakata K. Hip fracture incidence in Japan: estimates of new patients in 2007 and 20-year trends. Arch Osteoporos (2009) 4:71–7. 10.1007/s11657-009-0031-y 20234789 PMC2836738

[14] Lau EM Lee JK Suriwongpaisal P Saw SM Das De S Khir A The incidence of hip fracture in four Asian countries: the Asian Osteoporosis Study (AOS). Osteoporos Int (2001) 12:239–43. 10.1007/s001980170135 11315243

[15] Yan L Zhou B Prentice A Wang X Golden MH. Epidemiological study of hip fracture in Shenyang, People’s Republic of China. Bone (1999) 24:151–5. 10.1016/S8756-3282(98)00168-9 9951786

[16] Looker AC Wahner HW Dunn WL Calvo MS Harris TB Heyse SP Updated data on proximal femur bone mineral levels of US adults. Osteoporos Int (1998) 8:468–89. 10.1007/s001980050093 9850356

[17] Shin MH Zmuda JM Barrett-Connor E Sheu Y Patrick AL Leung PC Race/ethnic differences in associations between bone mineral density and fracture history in older men. Osteoporos Int (2014) 25:837–45. 10.1007/s00198-013-2503-6 24146094 PMC4058886

[18] Marshall LM Zmuda JM Chan BK Barrett-Connor E Cauley JA Ensrud KE Race and ethnic variation in proximal femur structure and BMD among older men. J Bone Miner Res (2008) 23:121–30. 10.1359/jbmr.070908 17892375 PMC2663587

[19] Pye S Ward K Adams J Finn J Wu F O’Neill T Influence of ethnicity on bone mineral density and HIP axis length in UK men. In: Society BR, editor. Bone Research Society/British Orthopaedic Research Society – Joint Meeting. Oxford: Frontiers Media SA (2013). 96 p.

[20] Ward K Jeffery M Pye S Adams J Boonen S Vanderschueren D Abstracts of the osteoporosis conference 2010. November 28-December 1, 2010. Liverpool, United Kingdom. Osteoporos Int (2010) 21:S443–518 10.1007/s00198-010-1388-x 20972669

[21] Mehta G Taylor P Petley G Dennison EM Cooper C Walker-Bone K. Bone mineral status in imigrant Indo-Asian women. Q J Med (2004) 97:97–9. 10.1093/qjmed/hch017 14747624

[22] Roy D Swarbrick C King Y Pye S Adams J Berry J Differences in peak bone mass in women of European and South Asian origin can be explained by differences in body size. Osteoporos Int (2005) 16:1254–62. 10.1007/s00198-005-1837-0 15702264

[23] Yan L Crabtree NJ Reeve J Zhou B Dequeker J Nijs J Does hip strength analysis explain the lower incidence of hip fracture in the People’s Republic of China? Bone (2004) 34:584–8. 10.1016/j.bone.2003.12.005 15003807

[24] Aspray TJ Prentice A Cole TJ Sawo Y Reeve J Francis RM. Low bone mineral content is common but osteoporotic fractures are rare in elderly rural Gambian women. J Bone Miner Res (1996) 11:1019–25. 10.1002/jbmr.5650110720 8797124

[25] Aspray TJ Prentice A Cole TJ. The bone mineral content of weight-bearing bones is influenced by the ratio of sitting to standing height in elderly Gambian women. Bone (1995) 17:261–3. 10.1016/8756-3282(95)98407-E 8541139

[26] Schnitzler CM Pettifor JM Mesquita JM Bird MD Schnaid E Smyth AE. Histomorphometry of iliac crest bone in 346 normal black and white South African adults. Bone Miner (1990) 10:183–99. 10.1016/0169-6009(90)90261-D 2224205

[27] Danielson ME Beck TJ Lian Y Karlamangla AS Greendale GA Ruppert K Ethnic variability in bone geometry as assessed by hip structure analysis: findings from the hip strength across the menopausal transition study. J Bone Miner Res (2013) 28:771–9. 10.1002/jbmr.1781 23044816 PMC3586935

[28] Dibba B Prentice A Laskey MA Stirling DM Cole TJ. An investigation of ethnic differences in bone mineral, hip axis length, calcium metabolism and bone turnover between West African and Caucasian adults living in the United Kingdom. Ann Hum Biol (1999) 26:229–42. 10.1080/030144699282732 10355494

[29] Prentice A Shaw J Laskey MA Cole TJ Fraser DR. Bone mineral content of British and rural Gambian women aged 18-80+ years. Bone Miner (1991) 12:201–14. 10.1016/0169-6009(91)90033-V 2021710

[30] Wang Q Teo JW Ghasem-Zadeh A Seeman E. Women and men with hip fractures have a longer femoral neck moment arm and greater impact load in a sideways fall. Osteoporos Int (2009) 20:1151–6. 10.1007/s00198-008-0768-y 18931818

[31] Duboeuf F Hans D Schott AM Kotzki PO Favier F Marcelli C Different morphometric and densitometric parameters predict cervical and trochanteric hip fracture: the EPIDOS Study. J Bone Miner Res (1997) 12:1895–902. 10.1359/jbmr.1997.12.11.1895 9383694

[32] Ward KA Roy DK Pye SR O’Neill TW Berry JL Swarbrick CM Forearm bone geometry and mineral content in UK women of European and South-Asian origin. Bone (2007) 41:117–21. 10.1016/j.bone.2007.03.013 17493888

[33] Ward K. Musculoskeletal phenotype through the life course: the role of nutrition. Proc Nutr Soc (2012) 71:27–37. 10.1017/S0029665111003375 22137032

[34] Darling AL Hakim OA Horton K Gibbs MA Cui L Berry JL Adaptations in tibial cortical thickness and total volumetric bone density in postmenopausal South Asian women with small bone size. Bone (2013) 55:36–43. 10.1016/j.bone.2013.03.006 23531785

[35] Liu XS Walker MD McMahon DJ Udesky J Liu G Bilezikian JP Better skeletal microstructure confers greater mechanical advantages in Chinese-American women versus white women. J Bone Miner Res (2011) 26:1783–92. 10.1002/jbmr.378 21351150 PMC3551974

[36] Walker MD Liu XS Stein E Zhou B Bezati E McMahon DJ Differences in bone microarchitecture between postmenopausal Chinese-American and white women. J Bone Miner Res (2011) 26:1392–8. 10.1002/jbmr.352 21305606 PMC3558983

[37] Putman MS Yu EW Lee H Neer RM Schindler E Taylor AP Differences in skeletal microarchitecture and strength in African-American and white women. J Bone Miner Res (2013) 28:2177–85. 10.1002/jbmr.1953 23572415 PMC3779478

[38] Baker JF Davis M Alexander R Zemel BS Mostoufi-Moab S Shults J Associations between body composition and bone density and structure in men and women across the adult age spectrum. Bone (2013) 53:34–41. 10.1016/j.bone.2012.11.035 23238122 PMC3552077

[39] De Laet C Kanis JA Oden A Johanson H Johnell O Delmas P Body mass index as a predictor of fracture risk: a meta-analysis. Osteoporos Int (2005) 16:1330–8. 10.1007/s00198-005-1863-y 15928804

[40] Nelson DA Beck TJ Wu G Lewis CE Bassford T Cauley JA Ethnic differences in femur geometry in the women’s health initiative observational study. Osteoporos Int (2011) 22:1377–88. 10.1007/s00198-010-1349-4 20737265

[41] Ho-Pham LT Nguyen UD Nguyen TV. Association between lean mass, fat mass, and bone mineral density: a meta-analysis. J Clin Endocrinol Metab (2014) 99:30–8. 10.1210/jc.2013-3190 24384013

[42] Compston JE Watts NB Chapurlat R Cooper C Boonen S Greenspan S Obesity is not protective against fracture in postmenopausal women: GLOW. Am J Med (2011) 124:1043–50. 10.1016/j.amjmed.2011.06.013 22017783 PMC4897773

[43] Premaor MO Pilbrow L Tonkin C Parker RA Compston J Obesity and fractures in postmenopausal women. J Bone Miner Res (2010) 25:292–7 10.1359/jbmr.091004 19821769

[44] Finkelstein JS Lee ML Sowers M Ettinger B Neer RM Kelsey JL Ethnic variation in bone density in premenopausal and early perimenopausal women: effects of anthropometric and lifestyle factors. J Clin Endocrinol Metab (2002) 87:3057–67. 10.1210/jcem.87.7.8654 12107201

[45] Nam HS Kweon SS Choi JS Zmuda JM Leung PC Lui LY Racial/ethnic differences in bone mineral density among older women. J Bone Miner Metab (2013) 31:190–8. 10.1007/s00774-012-0402-0 23143509 PMC4109723

[46] Prentice A Parsons T Cole T. Uncritical use of bone mineral density in absorptiometry may lead to size-related artifacts in the identification of bone mineral determinants. Am J Clin Nutr (1994) 60:837–42. 7985621 10.1093/ajcn/60.6.837

[47] Frost H Bone “mass” and the “mechanostat”: a proposal. Anat Rec (1987) 219:1–9 10.1002/ar.1092190104 3688455

[48] Rittweger J Schiessl H Felsenberg D Runge M. Reproducibility of the jumping mechanography as a test of mechanical power output in physically competent adult and elderly subjects. J Am Geriatr Soc (2004) 52:128–31. 10.1111/j.1532-5415.2004.52022.x 14687327

[49] Jeffrey M Age-related change and ethnic differences in neuromuscular function. 8th International Workshop For Musculoskeletal & Neuronal Interactions. Ipswich (2012).

[50] Khan UI Wang D Sowers MR Mancuso P Everson-Rose SA Scherer PE Race-ethnic differences in adipokine levels: the study of women’s health across the nation (SWAN). Metabolism (2012) 61:1261–9. 10.1016/j.metabol.2012.02.005 22444780 PMC3404256

[51] McKeigue PM Pierpoint T Ferrie JE Marmot MG. Relationship of glucose intolerance and hyperinsulinaemia to body fat pattern in south Asians and Europeans. Diabetologia (1992) 35:785–91. 1511807 10.1007/BF00429101

[52] Rush EC Freitas I Plank LD. Body size, body composition and fat distribution: comparative analysis of European, Maori, Pacific Island and Asian Indian adults. Br J Nutr (2009) 102:632–41. 10.1017/S0007114508207221 19203416

[53] Taaffe DR Cauley JA Danielson M Nevitt MC Lang TF Bauer DC Race and sex effects on the association between muscle strength, soft tissue, and bone mineral density in healthy elders: the health, aging, and body composition study. J Bone Miner Res (2001) 16:1343–52. 10.1359/jbmr.2001.16.7.1343 11450711

[54] Miljkovic I Cauley JA Petit MA Ensrud KE Strotmeyer E Sheu Y Greater adipose tissue infiltration in skeletal muscle among older men of African ancestry. J Clin Endocrinol Metab (2009) 94:2735–42. 10.1210/jc.2008-2541 19454588 PMC2730872

[55] Nam HS Shin MH Zmuda JM Leung PC Barrett-Connor E Orwoll ES Race/ethnic differences in bone mineral densities in older men. Osteoporos Int (2010) 21:2115–23. 10.1007/s00198-010-1188-3 20204598 PMC2974925

[56] Chantler S Dickie K Goedecke JH Levitt NS Lambert EV Evans J Site-specific differences in bone mineral density in black and white premenopausal South African women. Osteoporos Int (2012) 23:533–42. 10.1007/s00198-011-1570-9 21369790

[57] Malan NT Hamer M Schutte AE Huisman HW Van Rooyen JM Schutte R Low testosterone and hyperkinetic blood pressure responses in a cohort of South African men: the SABPA study. Clin Exp Hypertens (2013) 35:228–35. 10.3109/10641963.2012.721839 22994902

[58] Heo M Faith MS Pietrobelli A Heymsfield SB. Percentage of body fat cutoffs by sex, age, and race-ethnicity in the US adult population from NHANES 1999-2004. Am J Clin Nutr (2012) 95:594–602. 10.3945/ajcn.111.025171 22301924

